# SARS-CoV-2 and Immunity: Natural Infection Compared with Vaccination

**DOI:** 10.3390/ijms23168982

**Published:** 2022-08-11

**Authors:** Simone Vespa, Pasquale Simeone, Giulia Catitti, Davide Buca, Domenico De Bellis, Laura Pierdomenico, Damiana Pieragostino, Ilaria Cicalini, Piero Del Boccio, Luca Natale, Trevor Owens, Reza Khorooshi, Vincenzo De Laurenzi, Liborio Stuppia, Paola Lanuti

**Affiliations:** 1 Department of Medicine and Aging Sciences, University “G. d’Annunzio” of Chieti-Pescara, 66100 Chieti, Italy; 2 Center for Advanced Studies and Technology (CAST), University “G. d’Annunzio” of Chieti-Pescara, 66100 Chieti, Italy; 3 Department of Innovative Technologies in Medicine and Dentistry, University “G. d’Annunzio” of Chieti-Pescara, 66100 Chieti, Italy; 4 Department of Pharmacy, University “G. d’Annunzio” of Chieti-Pescara, 66100 Chieti, Italy; 5 Department of Neurobiology Research, Institute of Molecular Medicine, University of Southern Denmark, 5000 Odense, Denmark; 6 Department of Psychological, Health and Territory Sciences, School of Medicine and Health Sciences, University “G. d’Annunzio” of Chieti-Pescara, 66100 Chieti, Italy

**Keywords:** COVID-19 vaccines, T-cell polyfunctionality, polychromatic flow cytometry

## Abstract

Recently, the protective and/or pathological role of virus-specific T cells in SARS-CoV-2 infection has been the focus of many studies. We investigated the anti-spike IgG levels and SARS-CoV-2-specific T cells in 125 donors (90 vaccinated with four different vaccine platforms, 16 individuals with a previous natural infection, and 19 not vaccinated donors who did not report previous SARS-CoV-2 infections). Our data show that anti-spike IgG titers were similar between naturally infected subjects and those vaccinated with adenoviral vector vaccines. Of note, all immunized donors produced memory CD4^+^ and/or CD8^+^ T cells. A sustained polyfunctionality of SARS-CoV-2-specific T cells in all immunized donors was also demonstrated. Altogether, our data suggest that the natural infection produces an overall response like that induced by vaccination. Therefore, this detailed immunological evaluation may be relevant for other vaccine efforts especially for the monitoring of novel vaccines effective against emerging virus variants.

## 1. Introduction

During the last 2 years, the development of COVID-19 vaccines has been extremely fast and successful, and several manufacturers have obtained clinical authorization in a short period of time. These vaccines have been used worldwide in mass immunization programs; they have been largely demonstrated to prevent serious disease and death, and, in general, they have been fundamental in fighting the pandemic [1,2,3,4,5]. All currently authorized vaccines rely on the viral spike protein (S) as an immunogen [6], given that it is exposed on the surface of the virus and plays a crucial role in the recognition of human host cell surface receptor angiotensin-converting enzyme 2 (ACE2). This recognition is required for the fusion of viral and host cell membranes, and the transfer of the viral nucleocapsid into the host cells [7].

In Italy, two different mRNA vaccines, Moderna mRNA-1273 [8] and Pfizer/BioNTech BNT162b2 [9,10], and two viral vector-based vaccines, Janssen/J&J Ad26.COV2.S [11] and Oxford/AstraZeneca ChAdOx1-S [12], have been widely administered. These vaccines proved relevant efficacy against COVID-19 cases. Antibody levels are clearly related to the protection against virus infection during the first months post-vaccination [13,14]. Several studies, however, as expected, also underlined the relevance of T- and B-cell memory in protective immunity [15], with neutralizing antibodies playing a dominant role in infection prevention, cellular immunity modulating disease severity, and resolving SARS-CoV-2 infection [16]. Overall, available data suggest that the coordinated presence of all the components of the adaptive immunity is linked with protective immunity against COVID-19 [17]. It has also been demonstrated that a correct lifestyle (healthy diet and the correct balance of the gut microbiota) enhances the immune system performance [18]. On the other hand, uncoordinated activation of humoral and cellular immunity was observed in elderly individuals, who more often develop severe pathological consequences [17]. There are many publications regarding anti-spike IgG titers and T cells in SARS-CoV-2 infection, but comparisons in terms of phenotypes and functions of antigen-specific T cells induced by different COVID-19 vaccines are scarcely available. The relative contribution of humoral and cellular immunity in the protection process is still difficult to decipher. While a prediction of antibody levels required to induce protection has been proposed [19,20], the role of T cells is still difficult to analyze since the evaluation of virus-specific T cells is technically more complex than serological analyses. The massive COVID-19 vaccination campaign, therefore, represents a unique opportunity to study immune responses. Therefore, taking into account that antibodies and memory T cells, both CD4^+^ and CD8^+^, are, in some ways, all involved in protective immunity against COVID-19, we assessed the amplitude and the characteristics of vaccine-induced antibodies and immune T-cell memory with four different vaccine platforms, as well as the level of protection induced by the different vaccines.

## 2. Results

### 2.1. Spike Antibody Magnitude

**Figure 1:**
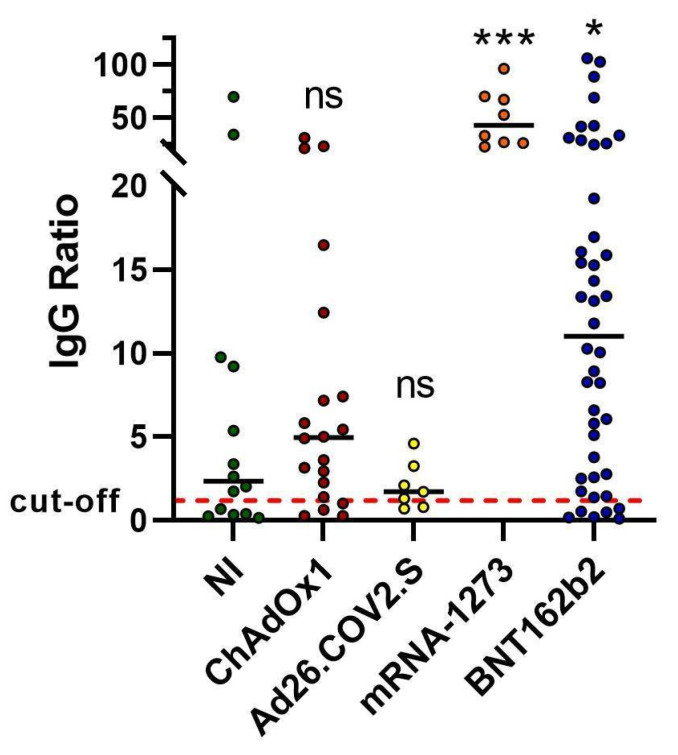
Antibodies elicited by resolved natural infections and COVID-19 vaccines. Comparison of longitudinal SARS-CoV-2 spike IgG levels among resolved natural infections (NI) and ChAdOx1, Ad26.COV2.S, mRNA-1273, and BNT162b2 COVID-19 vaccines were carried out. Individual data points are represented as scatter dot plots with lines showing the median value (cutoff value = 1.2). Statistical significance was calculated using nonparametric Mann–Whitney *U*-tests (two-tailed). A *p*-value <0.05 was considered statistically significant [ns (*p* > 0.05), * (*p* < 0.05), *** (*p* < 0.0005)].

For all donors, SARS-CoV-2 spike antibodies were measured when T-cell memory analyses were carried out (Figure 1). For ChAdOx1 vaccination, 80% of donors had detectable levels of spike IgG (>1.2). For Ad26.COV2.S, 71% of vaccinated donors displayed levels of spike IgG higher than the threshold, while, for mRNA-1273 and BNT162b2, 100% and 86% of individuals showed detectable levels of spike IgG, respectively (Figure 1). Interestingly, antibody titers of resolved natural infection subjects were similar to those produced in subjects vaccinated with adenoviral vector vaccines, and subjects vaccinated with mRNA vaccines displayed significantly higher levels of IgG (Figure 1).

### 2.2. Induction of Spike Specific CD4^+^ T Cells

**Figure 2:**
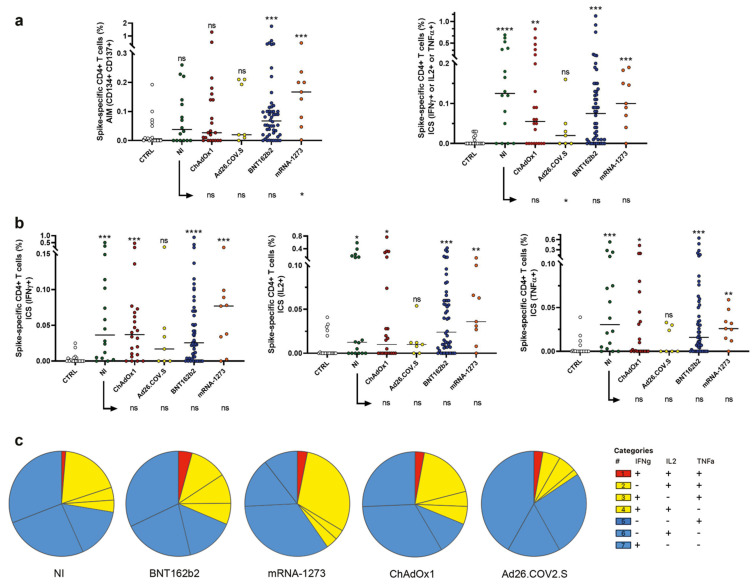
AIM, ICS, and polyfunctionality of spike-specific CD4^+^ T cells. Frequencies of CD4^+^ T cells expressing T-cell activation markers (**a**) and producing antigen-specific cytokines (**b**) are shown in SARS-CoV-2-unexposed healthy donors who never received any anti-SARS-CoV-2 vaccine (CTRL), in resolved natural infections (NI), and in donors vaccinated with two doses of ChAdOx1, a single dose of Ad26.COV2.S, and two doses of BNT162b2 and mRNA-1273 vaccines. Frequencies were obtained by subtracting to the values of the stimulated samples the background produced by the related unstimulated tube. Individual data points are represented as scatter dot plots with lines showing the median value. (**c**) Pie charts show the expression of cytokine combinations from CD4^+^ T cells following spike stimulation. Significance was calculated using nonparametric Mann–Whitney *U*-tests (two-tailed). A *p*-value <0.05 was considered statistically significant. [ns (*p* > 0.05), * (*p* < 0.05), ** (*p* < 0.005), *** (*p* < 0.0005), **** (*p* < 0.0001)].

The SARS-CoV-2 spike-specific CD4^+^ T-cell response was evaluated in all recruited subjects, using the previously described flow cytometry TCR-dependent activation-induced marker (AIM) assay (CD134 and CD137) combined with intracellular staining (ICS) for cytokines (IFN-γ, TNF-α, and IL-2) (Figure 2) [21,22]. The frequency of AIM^+^ (CD134^+^/CD137^+^) CD4^+^ T cells was evaluated. AIM^+^ memory CD4^+^ T cells were detectable in 89% of mRNA-vaccinated donors, in 72% of volunteers vaccinated with viral vector-based vaccines, and in 63% of NI subjects. Furthermore, as shown in Figure 2a, subjects vaccinated with the mRNA-1273 vaccine displayed higher frequencies of AIM^+^ CD4^+^ memory T cells than NI donors (*p* = 0.031).

When ICS data were analyzed, total frequencies of cells producing at least one of the tested cytokines (IFN-γ, TNF-α, and IL-2) were calculated for CD4^+^ spike-specific T cells. As shown in Figure 2b, CD4^+^ T cells producing at least one of the analyzed cytokines were detectable in 80% of mRNA-vaccinated donors, in 62% of volunteers vaccinated with viral vector-based vaccines, and in 75% of NI subjects. No differences in terms of total cytokine frequencies were detected between vaccinated individuals and donors with a resolved SARS-CoV-2 infection (Figure 2b).

We also aimed to analyze the polyfunctionality of SARS-CoV-2 spike-specific CD4^+^ T cells (Figure 2c). We defined polyfunctional populations as cells exhibiting positivity for ≥2 effector functions. We observed an overlap between the frequency of polyfunctional cells produced by SARS-CoV-2-specific CD4^+^ T cells in NI and ChAdOx1-vaccinated donors. Higher frequencies of polyfunctional SARS-CoV-2-specific CD4^+^ T cells were observed in donors vaccinated with mRNA-based vaccines.

### 2.3. Evaluation of Spike-Specific CD8^+^ T Cells

**Figure 3:**
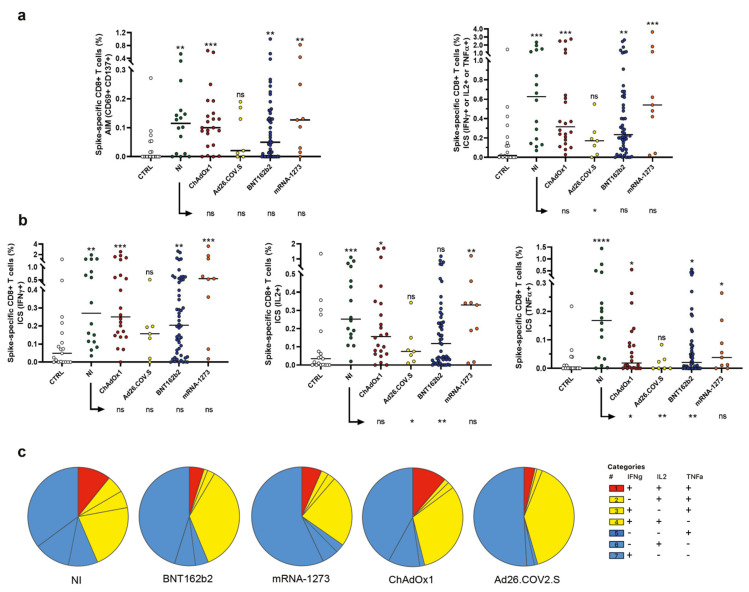
AIM and ICS of spike-specific CD8^+^ T cells. Frequencies of CD8^+^ T cells expressing T-cell activation markers (**a**) and producing antigen-specific cytokines (**b**) are shown in SARS-CoV-2 unexposed healthy donors who never received any anti-SARS-CoV-2 vaccine (CTRL), in resolved natural infections (NI), and in donors vaccinated with two doses of ChAdOx1, a single dose of Ad26.COV2.S, and two doses of BNT162b2 and mRNA-1273 vaccines. Frequencies were obtained by subtracting from the values of the stimulated samples the background produced by the related unstimulated tube. Individual data points are represented as scatter dot plots with lines showing the median value. (**c**) Pie charts show the expression of cytokine combinations from CD8^+^ T cells following spike stimulation. Significance was calculated using nonparametric Mann–Whitney *U*-tests (two-tailed). A *p*-value <0.05 was considered statistically significant. [ns (*p* > 0.05), * (*p* < 0.05), ** (*p* < 0.005), *** (*p* < 0.0005), **** (*p* < 0.0001)].

The SARS-CoV-2 spike-specific CD8^+^ T-cell response was evaluated using the AIM assay (CD69, CD137) combined with ICS for cytokines (IFN-γ, TNF-α, and IL-2) (Figure 3) [21,22]. AIM^+^ (CD69^+^/CD137^+^) CD8^+^ T cells were detectable in 74% of mRNA-vaccinated donors, in 86% of volunteers vaccinated with viral vector-based vaccines, and in 81% of resolved NI subjects. Apart from Ad26.COV2.S-vaccinated individuals that displayed lower frequencies of memory CD8^+^ T cells (Figure 3a right panel), comparable percentages of CD69^+^/CD137^+^/CD8^+^ T cells were observed in NI and vaccinated donors (Figure 3a, left panel). SARS-CoV-2 spike-specific CD8^+^ T cells were analyzed by ICS and stimulation-induced levels of IFN-γ, TNF-α, and IL-2 evaluated (CD69^+^/cytokine^+^ cells were identified as ICS^+^ cells) in NI donors, as well as in vaccinated subjects. CD8^+^ T cells producing at least one of the analyzed cytokines were detectable in 92% of mRNA-vaccinated donors, in 97% of volunteers vaccinated with viral vector-based vaccines, and in 100% of resolved NI subjects (Figure 3b). When the compartment of CD8^+^ spike-specific T cells was compared between NI and vaccinated subjects, it emerged that CD8^+^ spike-specific T cells in NI patients produced higher levels of TNF-α with respect to all vaccinated donors (except for mRNA-1273 vaccinated subjects), as shown in Figure 3b. Furthermore, spike-specific CD8^+^ T cells in subjects vaccinated with BNT162b2 and Ad26.COV2.S produced lower levels of IL-2 as compared to donors who had resolved the NI. Overall, combining AIM and ICS data for CD4^+^ and CD8^+^ T cells, 100% of NI and vaccinated subjects demonstrated a detectable response to the S protein pool (not shown).

When the polyfunctionality of SARS-CoV-2-specific CD8^+^ T cells was analyzed, we observed an evident similarity in the frequency of polyfunctional cells producing three cytokines in NI and ChAdOx1-vaccinated donors (Figure 3c). This fraction of multifunctional cells was higher in NI and ChAdOx1-vaccinated donors than in other cohorts. Lower frequencies of polyfunctional SARS-CoV-2-specific CD8^+^ T cells were observed in donors vaccinated with mRNA-1273.

### 2.4. The Frequency of Naïve, Effector Memory, and Central Memory T Cells Impacts the SARS-CoV-2-Specific Responses

We carried out a phenotypic analysis of T cells, to study the naïve (TN), the central memory (TCM), the effector memory (TEM), and the effector memory RA (TEMRA) compartments, as reported in Section 4, using an already published gating strategy [23].

**Figure 4:**
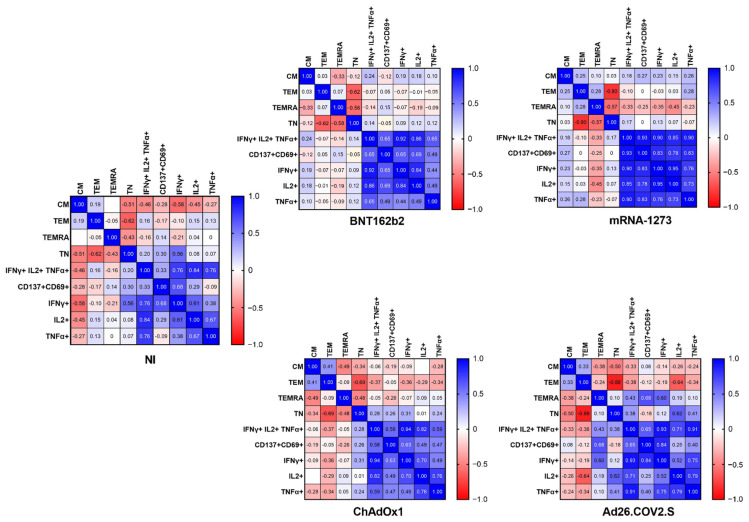
Correlation analysis between CD8^+^ T-cell subpopulations and produced cytokines. Correlation analysis was performed for NI and each vaccine platform. Each comparison shows a value representing the correlation strength between different sets of variables. Spearman’s rank correlation test was carried out to assess the correlation and calculate cutoff values. Values below or above the cutoff were considered statistically significant (*p*-value < 0.05) and reported as follows: NI: cutoff >+0.51; ChAdOx1: cutoff >+0.47; BNT162b2: cutoff >+0.33; mRNA-1273: cutoff >+0.73; Ad26.COV2.S: cutoff >+0.75.

A correlation analysis (Figure 4) demonstrated that, in vaccinated donors and in resolved NI volunteers, the naïve CD8^+^ T-cell compartment inversely correlated with effector memory T (TEM) cell frequencies. Furthermore, the naïve CD8^+^ T-cell compartment inversely correlated with TEMRA (TEMRA). The CD8^+^ TEMRA compartment also inversely correlated with T CD8^+^ central memory (CM) cells in BNT162b2 and ChAdOx1.

## 3. Discussion

Vaccines against COVID-19 have achieved relevant success in protecting individuals from severe symptoms. It is also clear that some limitations still exist, including differences in efficacy among viral-based and mRNA-based vaccines and natural infections. In such a context, it is largely known that the adaptive immune response, which is implicated in the control of most viral infections, and immune memory are central to the success of all vaccines [15]. It is, therefore, critical to study adaptive responses to SARS-CoV-2 and COVID-19 vaccines. Here, we compared different aspects of the adaptive responses to resolved NI, to two different mRNA vaccines (Moderna mRNA-1273, and Pfizer/BioNTech BNT162b2), and to two viral vector-based vaccines (Janssen/J&J Ad26.COV2.S and Oxford/AstraZeneca ChAdOx1-S).

Our serological data are consistent with previous reports [21,22,24] and show that antibody titers are comparable between NI and viral-based vaccines and higher in mRNA-based vaccines, suggesting that the vaccination produces similar or even better (mRNA-based vaccines) humoral protection than the NI.

In this study, we also demonstrated that CD4^+^ and/or CD8^+^ T-cell responses were detected in 100% of individuals with resolved NI, as well as in donors vaccinated with all four vaccines. Even if these findings are consistent with previously published data on COVID-19 vaccine T-cell responses [24], the data here reported expand these observations, also including NI and ChAdOx1-S evaluations, providing additional information on CD4^+^ and CD8^+^ T-cell subpopulations. Interestingly we observed that subjects vaccinated with mRNA-1273 display higher frequencies of CD4^+^ memory T cells than NI and other vaccinated individuals, suggesting a higher potential of such a vaccine to quickly initiate the adaptive immune response, thus reducing disease severity in a short time. We also show that all immunized groups of donors (NI and vaccinated subjects) display spike protein-reactive CD8^+^ T cells (CD69^+^/CD137^+^); furthermore, comparable frequencies of AIM^+^ CD8^+^ T cells were observed in NI and in donors vaccinated with mRNA vaccines or ChAdOx1. These data suggest that vaccination and NI are both successful in inducing the clearance of the virus, also guaranteeing better COVID-19 outcomes [17,25]. In line with previously reported data [15], we also observed that the percentages of cells producing effector function molecules, following a specific spike stimulation, were similar in NI and vaccinated donors, suggesting that the protection linked to the CD8 effector cells is similar if induced by NI or vaccination.

Of note, we analyzed the polyfunctionality of spike-specific T cells, observing that multifunctional CD4^+^ T cells were more frequent in mRNA-1273 immunization, while NI and ChAdOx1 induced comparable frequencies of polyfunctional CD4^+^ T cells. Whereas frequencies of polyfunctional CD8^+^ T cells were similar in NI subjects and ChAdOx1 immunized donors. It is known that generally, and specifically in SARS-CoV-2 infections, polyfunctional memory T cells are able to control viral infection more efficiently than monofunctional T cells [26]. Therefore, these data demonstrate that both NI and vaccine produce polyfunctional CD4^+^ and CD8^+^ T cells, able to be highly effective against the virus.

We also carried out a phenotypic analysis of T cells [23]. It has already been demonstrated that CD4^+^ T cells from COVID-19 patients do not show alterations in the frequency of naïve and effector/central memory populations [27], and our data confirmed these results (not shown). Instead, we demonstrated both for NI and, for the first time, for all types of vaccines that CD8^+^ T cells display a marked reduction in the frequency of CD8^+^ naïve T cells, with a parallel expansion of terminally differentiated CD197^−^CD45RA^−^ (TEM) and CD197^−^CD45RA^+^ TEMRA cells, possibly associated with an amplified cytotoxic function [23], which may, therefore, be associated with an effective immunosurveillance. Overall, our study demonstrates that all vaccines induce a spike-specific and polyfunctional T-cell response. The produced immune adaptive response is similar in both natural infection and vaccination, even if a slight advantage, in terms of frequencies of spike specific and polyfunctionality of CD4^+^ T cells, was observed after the administration of mRNA-1273. Those cells have been demonstrated to have features associated with protective immunity and give an advantage in terms of a rapid and effective antiviral response against recurrent infection by SARS-CoV-2 virus.

In this scenario, although neutralizing antibody responses are less efficient toward the new variants emerged since November 2021, T-cell responses are still effective and cross-reactive to some of the most frequent variants [28,29,30]. Even if a plethora of technologically different new vaccine platforms (i.e., plant-based COVID-19 vaccines) have been recently developed [31,32] and could induce different T-cell phenotypes and functions, these data are particularly relevant for further comparisons and demonstration of the success of those novel strategies, compared to natural immunization.

## 4. Materials and Methods

### 4.1. Patients

Peripheral blood samples from 125 volunteers were collected at the Center for Advanced Studies and Technology (CAST), University “G. d’Annunzio” of Chieti-Pescara, Italy. Informed consent was obtained for all recruited subjects (following point 32 of the Declaration of Helsinki 2013). For everyone, a peripheral blood sample was harvested following the Ethical Committee approval N.19 of 9 September 2021. The demographic characteristics of the enrolled cohort of volunteers are reported in Table S1. Among these donors, 90 received a complete cycle of vaccination (two doses of BNT162b2, *n* = 52, mRNA-1273, *n* = 9, and ChAdOx1-S, *n* = 22, and one dose of Ad26.COV2.S, *n* = 7, according to the accepted protocols), 16 subjects declared a resolved natural SARS-CoV-2 infection, and 19 donors were not vaccinated and did not report any previous infection (control group). No concomitant pathologies were declared by the enrolled donors. Given that it has been demonstrated that spike-specific T cells were already detectable at day 10 in good quantities [33], we recruited all donors 18 days ± 10 days after vaccination completion or infection resolution.

### 4.2. Anti-S1 Spike IgG Measurement

A fully automated solid-phase DELFIA (time-resolved fluorescence) immunoassay was used to measure the presence of IgG antibodies to SARS-CoV-2 in a few drops of blood, collected by finger-prick and dried on filter paper, using the GSP^®^/DELFIA^®^ Anti-SARS-CoV-2 IgG kit time-resolved fluoroimmunoassay on a GSP instrument (PerkinElmer, Mustionkatu, Turku, Finland). IgG levels were calculated as described, as a ratio of fluorescence of the sample over the calibrator [21,22,34,35]. The test was screened as positive subjects having IgG levels above the laboratory 1.2 cutoff [21].

### 4.3. PBMC Isolation, Stimulation, and Staining for Flow Cytometry Analysis

Peripheral blood mononuclear cells (PBMC) were stimulated as previously published [21,22], using a pool of spike peptides (PepTivator S, cat. 130-126-701, PepTivator S1, cat. 130-127-048, Peptivator S+, cat. 130-127-312, MiltenyiBiotec, Bergisch Gladbach, Germany) at the recommended concentrations for 16 h (37 °C, 5% of CO_2_), while negative control tubes were treated with the same amount of vehicle (DMSO) [21,22]. After 2 h of stimulation, samples were treated with 6.5 μL of GolgiStop (cat. 554724, BD Biosciences, La Jolla, CA, USA). The TCR-dependent activation-induced marker (AIM) and flow cytometry with intracellular cytokine staining (ICS) assays were carried out as reported [21,22]. Table S2 shows the reagent list for flow cytometry analyses. Data were analyzed using FlowJo v10.8.1 (BD Biosciences, La Jolla, CA, USA) and SPICE v 6.1 (provided by M. Roederer, National Institutes of Health) software. Background subtraction for polyfunctional analysis was performed using PESTLE v 2.0 software (provided by M. Roederer, National Institutes of Health). Functional subsets were obtained by “Boolean” gating [36]. Frequencies of T-cell responses were displayed as percentages of CD4^+^ or CD8^+^ T cells. T cells producing at least one of the tested cytokines in the CD4^+^ and CD8^+^ T-cell compartments were considered specific for S protein stimulation. Polyfunctional T cells were analyzed for NI and vaccinated individuals, but not for control subjects that did not show effector functions for polyfunctional purposes.

### 4.4. Gating Strategy

The gating strategy to detect SARS-CoV-2 S-reactive CD8^+^ and CD4^+^ T cells after their in vitro stimulation with SARS-CoV-2 S peptide pools is shown in Figure S1. As we already reported, after gating lymphocytes, singlets, live cells, CD3^+^ T and CD8^−^ (further identified as CD4^+^ cells) and CD8^+^ subsets, AIMs were identified for both CD4^+^ and CD8^+^ T cells using the corresponding dimethyl sulfoxide (DMSO) control to assess and subtract the background. Interferon (IFN)-γ, tumor necrosis factor (TNF)-α, and interleukin (IL)-2 were then individually analyzed for each subset (CD4^+^ and CD8^+^), using the corresponding DMSO control to assess and subtract the background [21,22]. A phenotypic analysis of the CD8^+^ T-cell compartment was also carried out (Figure S2). To this end, within the CD8^+^ T-cell population, T effector memory (TEM, CD197^−^/CD45RA^−^), T effector memory RA (TEMRA, CD197^−^/CD45RA^+^), T naïve (TN, CD197^+^/CD45RA^+^), and central memory (CM, CD197^+^/CD45RA^−^) compartments were identified, as already reported [23].

### 4.5. Statistical Analysis

Statistical analysis was performed using GraphPad Prism 9 (GraphPad Software, San Diego, CA USA). Pairwise comparisons between controls/natural infection and vaccines were analyzed using Mann–Whitney *U*-test (two-tailed). Statistical significance was denoted as follows: ns (*p* > 0.05), * (*p* < 0.05), ** (*p* < 0.005), *** (*p* < 0.0005), **** (*p* < 0.0001). Correlation analysis was carried out using Spearman’s rank correlation test.

## Data Availability

The authors declare that the data supporting the findings of this study are available within the paper and its supplementary information files. All datasets generated and analyzed are available from the corresponding author upon reasonable request.

## Supplementary Materials

The supporting information can be downloaded at: https://www.mdpi.com/article/10.3390/ijms23168982/s1.

## References

[1] Heinz F.X., Stiasny K. (2021). Distinguishing Features of Current COVID-19 Vaccines: Knowns and Unknowns of Antigen Presentation and Modes of Action. NPJ Vaccines.

[2] Hasan T., Beardsley J., Marais B.J., Nguyen T.A., Fox G.J. (2021). The Implementation of Mass-Vaccination against SARS-CoV-2: A Systematic Review of Existing Strategies and Guidelines. Vaccines.

[3] Haas E.J., Angulo F.J., McLaughlin J.M., Anis E., Singer S.R., Khan F., Brooks N., Smaja M., Mircus G., Pan K. (2021). Impact and Effectiveness of MRNA BNT162b2 Vaccine against SARS-CoV-2 Infections and COVID-19 Cases, Hospitalisations, and Deaths Following a Nationwide Vaccination Campaign in Israel: An Observational Study Using National Surveillance Data. Lancet.

[4] Rossman H., Shilo S., Meir T., Gorfine M., Shalit U., Segal E. (2021). COVID-19 Dynamics after a National Immunization Program in Israel. Nat. Med..

[5] Vasileiou E., Simpson C.R., Shi T., Kerr S., Agrawal U., Akbari A., Bedston S., Beggs J., Bradley D., Chuter A. (2021). Interim Findings from First-Dose Mass COVID-19 Vaccination Roll-out and COVID-19 Hospital Admissions in Scotland: A National Prospective Cohort Study. Lancet.

[6] Barnes C.O., Jette C.A., Abernathy M.E., Dam K.-M.A., Esswein S.R., Gristick H.B., Malyutin A.G., Sharaf N.G., Huey-Tubman K.E., Lee Y.E. (2020). SARS-CoV-2 Neutralizing Antibody Structures Inform Therapeutic Strategies. Nature.

[7] Jackson C.B., Farzan M., Chen B., Choe H. (2022). Mechanisms of SARS-CoV-2 Entry into Cells. Nat. Rev. Mol. Cell Biol..

[8] Jackson L.A., Anderson E.J., Rouphael N.G., Roberts P.C., Makhene M., Coler R.N., McCullough M.P., Chappell J.D., Denison M.R., Stevens L.J. (2020). An MRNA Vaccine against SARS-CoV-2—Preliminary Report. N. Engl. J. Med..

[9] Vogel A.B., Kanevsky I., Che Y., Swanson K.A., Muik A., Vormehr M., Kranz L.M., Walzer K.C., Hein S., Güler A. (2021). BNT162b Vaccines Protect Rhesus Macaques from SARS-CoV-2. Nature.

[10] Walsh E.E., Frenck R.W., Falsey A.R., Kitchin N., Absalon J., Gurtman A., Lockhart S., Neuzil K., Mulligan M.J., Bailey R. (2020). Safety and Immunogenicity of Two RNA-Based COVID-19 Vaccine Candidates. N. Engl. J. Med..

[11] Sadoff J., Gray G., Vandebosch A., Cárdenas V., Shukarev G., Grinsztejn B., Goepfert P.A., Truyers C., Fennema H., Spiessens B. (2021). Safety and Efficacy of Single-Dose Ad26.COV2.S Vaccine against COVID-19. N. Engl. J. Med..

[12] Falsey A.R., Sobieszczyk M.E., Hirsch I., Sproule S., Robb M.L., Corey L., Neuzil K.M., Hahn W., Hunt J., Mulligan M.J. (2021). Phase 3 Safety and Efficacy of AZD1222 (ChAdOx1 NCoV-19) COVID-19 Vaccine. N. Engl. J. Med..

[13] Gilbert P.B., Montefiori D.C., McDermott A.B., Fong Y., Benkeser D., Deng W., Zhou H., Houchens C.R., Martins K., Jayashankar L. (2022). Immune Correlates Analysis of the MRNA-1273 COVID-19 Vaccine Efficacy Clinical Trial. Science.

[14] Khoury D.S., Cromer D., Reynaldi A., Schlub T.E., Wheatley A.K., Juno J.A., Subbarao K., Kent S.J., Triccas J.A., Davenport M.P. (2021). Neutralizing Antibody Levels Are Highly Predictive of Immune Protection from Symptomatic SARS-CoV-2 Infection. Nat. Med..

[15] Sette A., Crotty S. (2021). Adaptive Immunity to SARS-CoV-2 and COVID-19. Cell.

[16] Keeton R., Richardson S.I., Moyo-Gwete T., Hermanus T., Tincho M.B., Benede N., Manamela N.P., Baguma R., Makhado Z., Ngomti A. (2021). Prior Infection with SARS-CoV-2 Boosts and Broadens Ad26.COV2.S Immunogenicity in a Variant-Dependent Manner. Cell Host Microbe.

[17] Rydyznski Moderbacher C., Ramirez S.I., Dan J.M., Grifoni A., Hastie K.M., Weiskopf D., Belanger S., Abbott R.K., Kim C., Choi J. (2020). Antigen-Specific Adaptive Immunity to SARS-CoV-2 in Acute COVID-19 and Associations with Age and Disease Severity. Cell.

[18] Rishi P., Thakur K., Vij S., Rishi L., Singh A., Kaur I.P., Patel S.K.S., Lee J.-K., Kalia V.C. (2020). Diet, Gut Microbiota and COVID-19. Indian J. Microbiol..

[19] Cromer D., Juno J.A., Khoury D., Reynaldi A., Wheatley A.K., Kent S.J., Davenport M.P. (2021). Prospects for Durable Immune Control of SARS-CoV-2 and Prevention of Reinfection. Nat. Rev. Immunol..

[20] Feng S., Phillips D.J., White T., Sayal H., Aley P.K., Bibi S., Dold C., Fuskova M., Gilbert S.C., Hirsch I. (2021). Correlates of Protection against Symptomatic and Asymptomatic SARS-CoV-2 Infection. Nat. Med..

[21] Lanuti P., Rossi C., Cicalini I., Pierdomenico L., Damiani V., Semeraro D., Verrocchio S., Del Boccio P., Evangelista A., Sarra A. (2021). Picture of the Favourable Immune Profile Induced by Anti-SARS-CoV-2 Vaccination. Biomedicines.

[22] Rossi C., Lanuti P., Cicalini I., De Bellis D., Pierdomenico L., Del Boccio P., Zucchelli M., Natale L., Sinjari B., Catitti G. (2021). BNT162b2 MRNA Vaccination Leads to Long-Term Protection from COVID-19 Disease. Vaccines.

[23] Mazzoni A., Salvati L., Maggi L., Capone M., Vanni A., Spinicci M., Mencarini J., Caporale R., Peruzzi B., Antonelli A. (2020). Impaired Immune Cell Cytotoxicity in Severe COVID-19 Is IL-6 Dependent. J. Clin. Investig..

[24] Zhang Z., Mateus J., Coelho C.H., Dan J.M., Moderbacher C.R., Gálvez R.I., Cortes F.H., Grifoni A., Tarke A., Chang J. (2022). Humoral and Cellular Immune Memory to Four COVID-19 Vaccines. Cell.

[25] Peng Y., Mentzer A.J., Liu G., Yao X., Yin Z., Dong D., Dejnirattisai W., Rostron T., Supasa P., Liu C. (2020). Broad and Strong Memory CD4+ and CD8+ T Cells Induced by SARS-CoV-2 in UK Convalescent Individuals Following COVID-19. Nat. Immunol..

[26] Jung J.H., Rha M.-S., Sa M., Choi H.K., Jeon J.H., Seok H., Park D.W., Park S.-H., Jeong H.W., Choi W.S. (2021). SARS-CoV-2-Specific T Cell Memory Is Sustained in COVID-19 Convalescent Patients for 10 Months with Successful Development of Stem Cell-like Memory T Cells. Nat. Commun..

[27] Mazzoni A., Salvati L., Maggi L., Annunziato F., Cosmi L. (2021). Hallmarks of Immune Response in COVID-19: Exploring Dysregulation and Exhaustion. Semin. Immunol..

[28] Thakur P., Thakur V., Kumar P., Singh Patel S.K. (2022). Emergence of Novel Omicron Hybrid Variants: BA(x), XE, XD, XF More than Just Alphabets. Int. J. Surg..

[29] Guo L., Wang G., Wang Y., Zhang Q., Ren L., Gu X., Huang T., Zhong J., Wang Y., Wang X. (2022). SARS-CoV-2-Specific Antibody and T-Cell Responses 1 Year after Infection in People Recovered from COVID-19: A Longitudinal Cohort Study. Lancet Microbe.

[30] Keeton R., Tincho M.B., Ngomti A., Baguma R., Benede N., Suzuki A., Khan K., Cele S., Bernstein M., Karim F. (2022). T Cell Responses to SARS-CoV-2 Spike Cross-Recognize Omicron. Nature.

[31] Karpiński T.M., Ożarowski M., Seremak-Mrozikiewicz A., Wolski H., Wlodkowic D. (2021). The 2020 Race towards SARS-CoV-2 Specific Vaccines. Theranostics.

[32] Hager K.J., Pérez Marc G., Gobeil P., Diaz R.S., Heizer G., Llapur C., Makarkov A.I., Vasconcellos E., Pillet S., Riera F. (2022). Efficacy and Safety of a Recombinant Plant-Based Adjuvanted COVID-19 Vaccine. N. Engl. J. Med..

[33] Kalimuddin S., Tham C.Y.L., Qui M., de Alwis R., Sim J.X.Y., Lim J.M.E., Tan H.-C., Syenina A., Zhang S.L., Le Bert N. (2021). Early T Cell and Binding Antibody Responses Are Associated with COVID-19 RNA Vaccine Efficacy Onset. Med.

[34] Cicalini I., Rossi C., Natale L., Cufaro M.C., Catitti G., Vespa S., De Bellis D., Iannetti G., Lanuti P., Bucci I. (2021). Passive Immunity to SARS-CoV-2 at Birth Induced by Vaccination in the First Trimester of Pregnancy. Int. J. Environ. Res. Public Health.

[35] Cicalini I., Del Boccio P., Zucchelli M., Rossi C., Natale L., Demattia G., De Bellis D., Damiani V., Tommolini M.L., Pizzinato E. (2022). Validation of the GSP^®^/DELFIA^®^ Anti-SARS-CoV-2 IgG Kit Using Dried Blood Samples for High-Throughput Serosurveillance and Standardized Quantitative Measurement of Anti-Spike S1 IgG Antibody Responses Post-Vaccination. Vaccines.

[36] Lanuti P., Ciccocioppo F., Bonanni L., Marchisio M., Lachmann R., Tabet N., Pierdomenico L., Santavenere E., Catinella V., Iacone A. (2012). Amyloid-Specific T-Cells Differentiate Alzheimer’s Disease from Lewy Body Dementia. Neurobiol. Aging.

