# Supplementary material for: SARS-CoV-2 and Immunity: Natural Infection Compared with Vaccination
Source: Int J Mol Sci. 2022 Aug 11;23(16):8982. doi: 10.3390/ijms23168982 (PMC9409314; doi:10.3390/ijms23168982)
Supplement: Supplementary file 1 [file ijms-23-08982-s001.zip › ijms-1862222-supplementary.pdf]

Supplementary Information

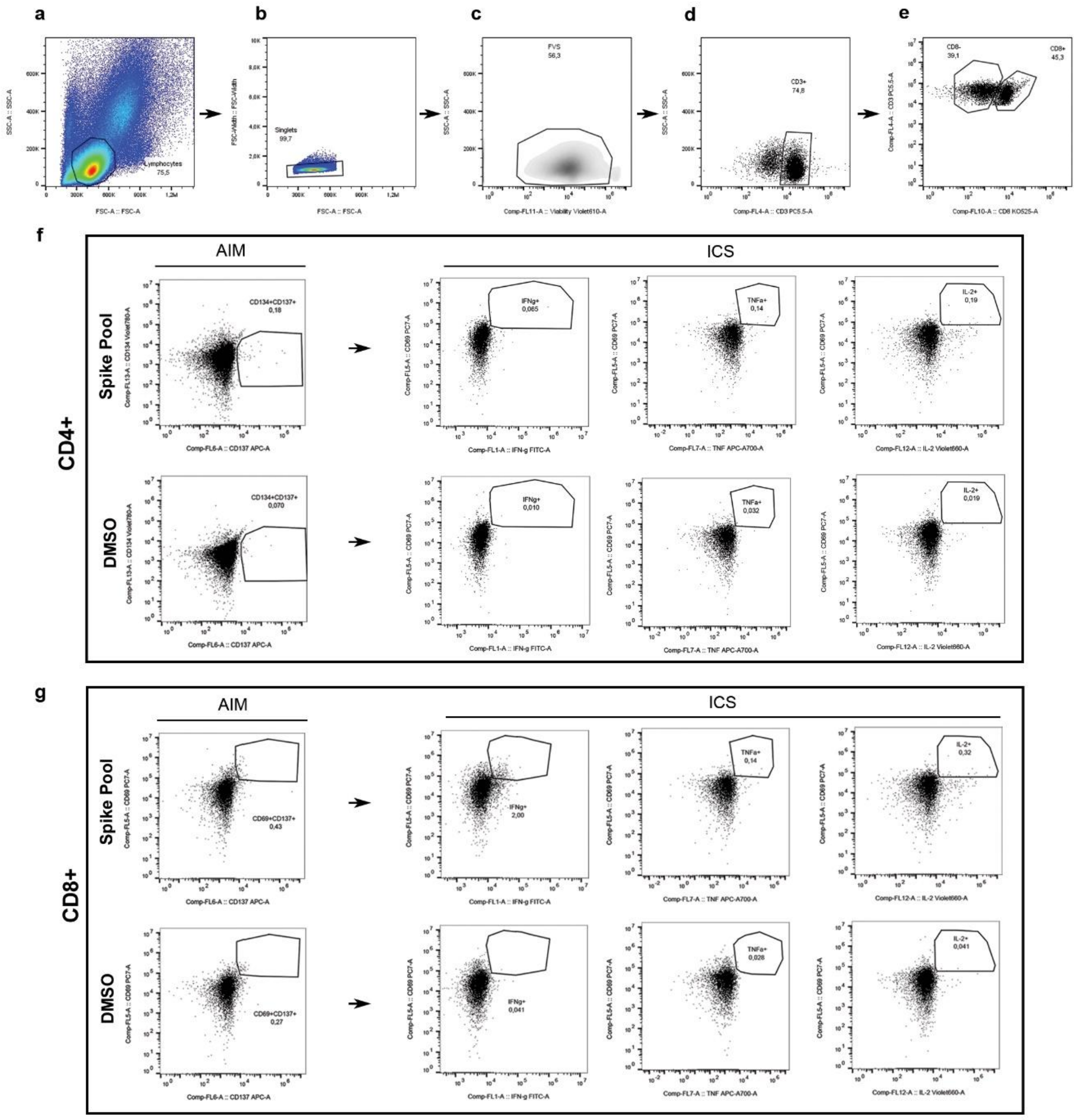

Figure S1. Gating strategy of SARS-CoV-2 spike-specific T lymphocytes. As a first step, lymphocytes were selected in a forward scatter (FSC-A)/side scatter (SSC-A) dot-plot (**a**). Then, aggregates were excluded (FSC-A/FSC-W dot-plot, **b**) and live cells were identified (FVS- events, **c**). Next, CD3+ T-cells were gated in a CD3/SSC-A dot-plot (**d**). Among T cells, CD8- (further identified as CD4+ cells) and CD8+ subsets were gated (**e**). TCR-dependent activation induced markers (AIM) and intracellular cytokine staining assays (ICS) were evaluated for both CD4+ and CD8+ T cells (**f**, **g**, respectively), using the corresponding dimethyl sulfoxide (DMSO) control to assess and subtract the background.

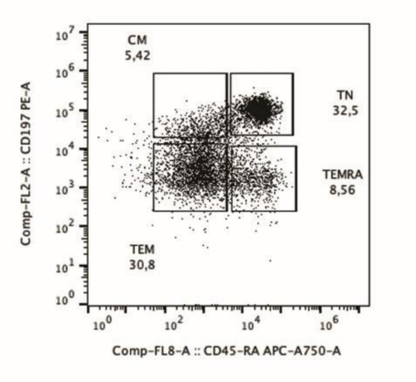

Figure S2. Phenotypic analysis of CD8+ T cells. Within the CD8+ T cell population (selected as shown in Figure S1 (A-E), T effector memory (TEM, CD197-/CD45RA-), T effector memory RA (TEMRA, CD197-/CD45RA+), T naïve (TN, CD197+/CD45RA+), and central memory compartments (CM, CD197+/CD45RA-) were gated.

**Supplementary Table S1**			
**Cohort**	**n**	**Age (years)**	**Age (median)**
**CTRL**	19	44.94 ± 11.43	44
**NI**	16	58.5 ± 12.85	62.5
**ChAdOx1**	22	46.76 ± 11.25	45
**Ad26.COV2.S**	7	46.28 ± 10.72	51
**BNT162b2**	52	48.15 ± 15.74	47.5
**mRNA-1273**	9	50.00 ± 12.67	53
**Gender**	**%**	**Age (years)**	**Age (median)**
**Male**	50.4	50.11 ± 14.71	50
**Female**	49.6	47.51 ± 13.15	45

Table S1. Demographic characteristics of the study participants. Displayed data represent mean ± standard deviation and the median age of subjects enrolled in the study from each vaccine platform.

Supplementary Table S2					
**SURFACE**					
Antigen	***Clone***	***Fluorochrome***	***Amount per test***	***Manufacturer***	***Cat. No***
CD197	2-L1-A	PE	5 µl	BD Biosciences	566742
CD3	SK7	PerCP-Cy5.5	20 µl	BD Biosciences	332771
CD69	FN50	PE-Cy7	5 µl	BD Biosciences	561928
CD45RA	HI100	APC-H7	5 µl	BD Biosciences	560674
CD8	SK1	V500-C	5 µl	BD Biosciences	647457
FVS 575V	NA	NA	1 µl	BD Biosciences	565694
**INTRACELLULAR**					
Antigen	***Clone***	***Fluorochrome***	***Amount per test***	***Manufacturer***	***Cat. No***
CD134	L106	BV768	5 µl	BD Biosciences	744746
CD137	4B4-1	APC	20 µl	BD Biosciences	550890
IFN-γ	B27	FITC	20 µl	BD Biosciences	552887
TNF-α	MAb11	Alexa Fluor™700	5 µl	BD Biosciences	557996
IL-2	5344.111	BV711	5 µl	BD Biosciences	563946

Table S2. Flow cytometry reagents. In the list are shown the antibodies utilized in the study for surface and intracellular staining. NA = not applicable.
